# CARFMAP: A Curated Pathway Map of Cardiac Fibroblasts

**DOI:** 10.1371/journal.pone.0143274

**Published:** 2015-12-16

**Authors:** Hieu T. Nim, Milena B. Furtado, Mauro W. Costa, Hiroaki Kitano, Nadia A. Rosenthal, Sarah E. Boyd

**Affiliations:** 1 Australian Regenerative Medicine Institute, Monash University, Clayton, VIC, 3800, Australia; 2 Faculty of Information Technology, Monash University, Clayton, VIC, 3800, Australia; 3 Laboratory for Disease Systems Modeling, RIKEN Center for Integrative Medical Sciences, Yokohama, Japan; 4 Okinawa Institute of Science and Technology, Onna, Onna-son, Kunigami, Okinawa, Japan; 5 National Heart and Lung Institute, Imperial College London, White City, W12 0NN, United Kingdom; 6 The Jackson Laboratory, Bar Harbor, ME, 04609, United States of America; Texas A & M University Health Science Center, UNITED STATES

## Abstract

The adult mammalian heart contains multiple cell types that work in unison under tightly regulated conditions to maintain homeostasis. Cardiac fibroblasts are a significant and unique population of non-muscle cells in the heart that have recently gained substantial interest in the cardiac biology community. To better understand this renaissance cell, it is essential to systematically survey what has been known in the literature about the cellular and molecular processes involved. We have built CARFMAP (http://visionet.erc.monash.edu.au/CARFMAP), an interactive cardiac fibroblast pathway map derived from the biomedical literature using a software-assisted manual data collection approach. CARFMAP is an information-rich interactive tool that enables cardiac biologists to explore the large body of literature in various creative ways. There is surprisingly little overlap between the cardiac fibroblast pathway map, a foreskin fibroblast pathway map, and a whole mouse organism signalling pathway map from the REACTOME database. Among the use cases of CARFMAP is a common task in our cardiac biology laboratory of identifying new genes that are (1) relevant to cardiac literature, and (2) differentially regulated in high-throughput assays. From the expression profiles of mouse cardiac and tail fibroblasts, we employed CARFMAP to characterise cardiac fibroblast pathways. Using CARFMAP in conjunction with transcriptomic data, we generated a stringent list of six genes that would not have been singled out using bioinformatics analyses alone. Experimental validation showed that five genes (*Mmp3*, *Il6*, *Edn1*, *Pdgfc* and *Fgf10*) are differentially regulated in the cardiac fibroblast. CARFMAP is a powerful tool for systems analyses of cardiac fibroblasts, facilitating systems-level cardiovascular research.

## Introduction

The adult mammalian heart contains many different cell types that work in unison under tightly regulated conditions to maintain normal cardiac function (*i*.*e*. homeostasis) [1]. Understanding homeostasis in the adult normal heart is critical as a baseline for unravelling the mechanisms behind heart diseases, especially those mechanisms that are unrelated to genetic defects. Cardiac fibroblasts (CFb) originate from several sources [2, 3], but despite comprising a significant proportion of heart non-muscle cells [4], the role of CFb in adult heart structure and function is still poorly understood [5, 6]. Within the adult heart, the CFb maintain tight electrical, chemical, and mechanical linkages to all other cardiac cells types [2], suggesting that this cell type may hold important structural and functional roles in heart homeostasis.

**Fig 1:**
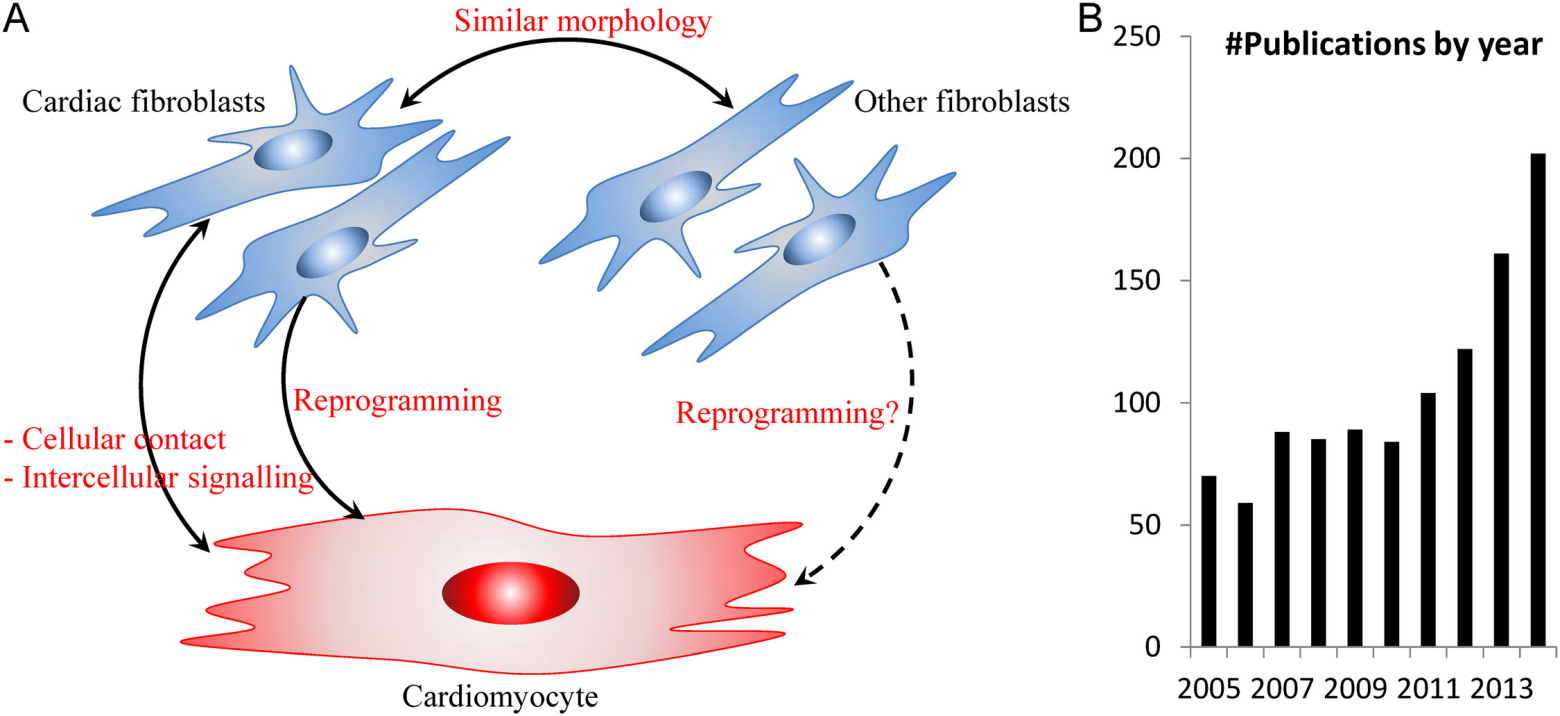
Rationale for mapping the cardiac fibroblast. (A) Relationship between cardiac fibroblast and other cell types. (B) Yearly publication in PubMed database related to the cardiac fibroblast. Both cardiac fibroblasts and fibroblasts from other sources (such as that from the tail) have been recently demonstrated to be capable of being reprogrammed to an induced cardiomyocyte-like state [8]. Question mark indicates further research is needed to fully understand both the reprogramming mechanisms and the cardiomyocyte identity.

The cardiac research community has traditionally attributed most critical heart functions to the heart muscle cells, the cardiomyocytes (CM). Morphologically and functionally, CFb are much more different from CM [1] compared to other fibroblasts such as from the tail (Fig 1A). Yet recent studies have demonstrated reprogramming of CFb to CM [7, 8]. Subsequently, Furtado *et al*. recently identified a set of transcription factors (TFs) that are unique to mouse CFb compared to tail fibroblasts (TFb) [9]. Among these genes are Gata4 and Tbx20, which are well-known to co-regulate important cardiac functions and structure, especially in CMs [10]. This led us and other peer cardiac biologists to rethink the role of the CFb, and view this cell type as much more specialised to its organ of origin. Indeed, the CFb literature has observed a rapid growth in the past five years (Fig 1B).

In a previous analysis [9], we manually identified the Tbx20 and Gata4 TFs in adult mouse CFb by applying our own specialised knowledge. However, these TFs were likely to only represent a subset of CFb genes that may be critical to heart homeostasis; less well-known genes may be of equal or potentially even greater biological importance. Thus we performed an unbiased, systems-level analysis to better understand the transcriptional activities in CFb cells. Such an analysis also helps put CFb transcriptional regulation into the context of the heart environment, where it may interact with other cells, especially the CM, to maintain heart homeostasis (Fig 1A).

We present here a new approach that produces a map of the CFb transcriptional network, which will promote a more systems-level understanding of the CFb, as well as the heterogeneous cardiac environment.

## Methods

### Animal ethics

All mice were kept in a full BL6-J background, housed at Monash Animal Services and experiments conformed with requirements under the ethics application MARP-2011-175. Mice were given standard housing conditions, in a 12h light/dark cycle with ad libitum food and water. Mice were monitored daily for clinical symptom or illness following standard operating protocols by Monash Animal Service, and none of the mice exhibited any clinical symptom or illness during these experiments. Mice were humanely euthanised via carbon dioxide inhalation. All experiments were conducted following guidelines from the National Health and Medical Research Council (NHMRC) and were approved by Monash Animal Research Platform Animal Ethics Committees (Monash University).

### Software implementation

CARFMAP was implemented using the NodeXL framework, which was built using the C# language as an add-in for Microsoft Excel^®^. The bio-entities and bio-relations were converted to the NodeXL syntax, which allows the CARFMAP to generate the nodes (bio-entities) and edges (bio-relations) in an interactive graph. The metadata associated with each bio-relation (organism, experiment type, *etc*.) were encoded into a graph attribute of the corresponding node, such as size or shape.

To achieve the graph layout of CARFMAP, we devised a custom layout algorithm (Box 1). In brief, the coordinates of every node were determined using a pair of values: radius and angle. Each class of node (drug, extracellular proteins, intracellular proteins, *etc*.) was assigned a specified radius value. At each given radius (class), the angle of each node was then calculated by dividing 360 degrees by the number of nodes in that radius, producing a symmetrical distribution as seen in CARFMAP. Finally, the computed (radius, angle) pair was converted into the standard Cartesian coordinate to layout the graph.

**Box 1. The CARFMAP custom layout algorithm tailored to literature-based pathway maps**

1. For each bio-entities *e*
  - If *e* is a “phenotype”
     radius(*e*) = 2
  - Else if *e* is a “gene expression”
     radius(*e*) = 3
  - Else if *e* is an “intracellular protein”
     radius(*e*) = random integer between 4 and 7, inclusive
  - Else if *e* is a “receptor”
     radius(*e*) = 8
  - Else if *e* is an “physiological condition”
     radius(*e*) = 9
  - Else if *e* is a “drug” or an “extracellular protein”
     radius(*e*) = 10
2. For each radius *r* value from 2 to 10
  - Compute S(*r*), the number of bio-entities at radius *r*
  - For the *i*th bio-entities *e* in radius r
     angle(*e*) = 360 / S(*r*) * *i*
3. For each bio-entities *e*, layout the node at coordinate (*x*, *y*), where
    x = radius(*e*) * cos(angle(*e*))
    y = radius(e) * sin(angle(e))

To improve human-readability, the algorithm assigned bio-entities into different concentric rings based on the node types.

### Text mining and automated text collection

The Boolean search term for retrieving articles related to the cardiac fibroblasts was:

[*“cardiac fibroblast” OR “cardiac fibroblasts” OR “heart fibroblast” OR “heart fibroblasts”*]

A straightforward search on PubMed for [cardiac fibroblast] would yield ~11,000 hits, but in the majority of cases, those articles would reference “cardiac” and “fibroblast” as separate terms, and not actually provide information about the cardiac fibroblast cell type. Therefore, in our Boolean search term, cardiac fibroblasts were put in quotes to avoid retrieving non-cardiac fibroblast articles. The above Boolean search term was input to the NCBI PubMed search field, with the “XML” option as output. For MEDIE, the search term was input to the “subject” field, with the other fields all left blank. For BioIE, the abstracts were written into a text file, and then uploaded to the server. For Whatizit, the list of PubMed IDs was input to the “Place your text/query here” text box. All results were collected as sentences with labelled terms (described in details in the Results section), which facilitated the manual curation process.

### Sample preparation, microarray and qPCR

Mouse cardiac and tail fibroblast preparation, microarray and RT-qPCR assays were performed as previously described [11]. Mouse primer for qPCR experiments are listed below:

*Edn1*: for 5’-GGCCCAAAGTACCATGCAGA; rev 5’-TGCTATTGCTGATGGCCTCC.

*Fgf10*: for 5’-GGAGATGTCCGCTGGAGAAG; rev 5’-CTGTTGATGGCTTTGACGGC.

*Il18*: for 5’-TCAGACAACTTTGGCCGACT; rev 5’-CAGTCTGGTCTGGGGTTCAC.

*Il6*: for 5’-CACGGCCTTCCCTACTTCAC; rev 5’-TGCAAGTGCATCATCGTTGT.

*Mmp3*: for 5’-AAGGGTGGATGCTGTCTTTGA; rev 5’-TGCCTTCCTTGGATCTCTTTTT.

*Pdgfc*: for 5’-TTAGGACGCTGGTGTGGTTC; rev 5’-ACCGAAGGACTCGTGGTTTC.

*Hprt*: for 5’- GCGAGGGAGAGCGTTGGGCT; rev 5’- CATCATCGCTAATCACGACGCTGGG.

### Bioinformatics analysis

The full R code to reproduce the bioinformatics analysis is available at http://visionet.erc.monash.edu/CARFMAP. In brief, raw single-channel signals provided by the Agilent Feature Extraction Software 11.0.1.1image analysis software were used for data analysis. Non-uniform, saturated probes, and population outliers were filtered using the default “Compromised” option in GeneSpring GX12.6 (Agilent), with threshold raw signal of 1.0. At the end of this process, 6 text files (.txt) were exported for the data normalisation stage.

Data normalisation was performed with R (http://www.r-project.org) using the publicly available Bioconductor packages (bioconductor.org) [12]. Three pre-processing and normalisation steps were performed: (1) use *read*.*maimage* function to extract the *gProcesedSignal* values from the GeneSpring exported data files; (2) use *avereps*.*EList* function to average the duplicate spots and log-2 transformation; and (3) use *normalizeBetweenArrays* function to perform quantile normalisation on all arrays.

Differential analysis between CFb and TFb samples was performed using the Bioconductor LIMMA package [13], which applies linear models and differential expression functions to the transcriptomic data. With 6 normalised arrays having identical distributions, the *lmFit* function identifies the genes that have differential expression between 3 CFb samples and 3 TFb samples. At p-value threshold of 0.05, we identified a pool of 3924 differentially expressed entities and these entities were used for fold-change calculation. The fold-changes were converted to numerical colour values (from red to yellow) for CARFMAP visualisation.

## Results

### Literature data collection and curation

**Fig 2:**
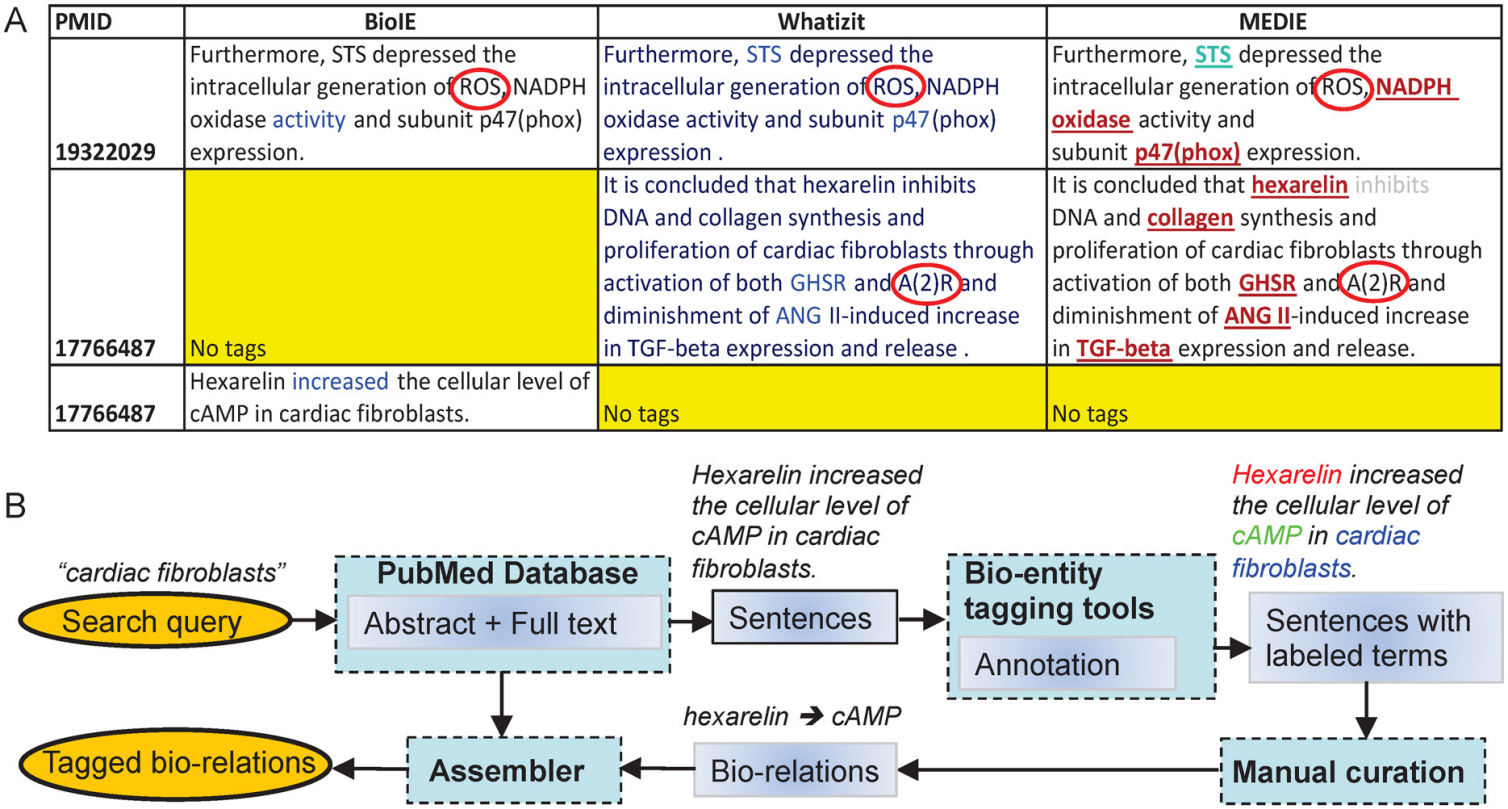
The hybrid automated-manual text mining pipeline. (A) The three most powerful automated biomedical-tagging engines, BioIE, Whatizit and MEDIE have specific limitations. BioIE only tags relationships between biomedical entities, and Whatizit only tags bio-entities. MEDIE tags both bio-entities and their relationships. In the given example, all three search engines failed to tag the bio-entities “*ROS*” and “*A(2)R*” (circled), which are obvious to a human reader. Red circles denote terms that the automated text mining algorithms failed to recognise. (B) The hybrid data processing pipeline combines automated text mining (BioIE, Whatizit and MEDIE) and manual text collection. Bio-entities are annotated with BioIE, Whatizit, MEDIE and PubTator.

The scientific interest in CFb has accelerated noticeably in the past 5 years, judged by the yearly number of publications about CFb in PubMed (Fig 1B). To evaluate the literature with the broadest coverage in an unbiased manner, researchers can adopt two main text mining approaches: automated algorithm-based text mining (reviewed by Rebholz-Schuhman [14]), and manual curation (exemplified by Caron et al. [15]). Algorithm-based text mining has been shown to be increasingly powerful and rapidly maturing, exhibiting respectably high prediction accuracy in network biology [16]. However, manual curation is still regarded as the most accurate approach for literature data collection, albeit with the serious drawback of requiring massive manpower [17]. To follow the standard terminology of the field of literature text mining, in this paper we refer to “bio-entities” as all biologically relevant terms (genes, proteins, drugs, *etc*.) and “bio-relations” as the relationships between the bio-entities (inhibits, activates, *etc*.). We performed algorithm-based text mining by combining three standard tools: MEDIE (http://www.nactem.ac.uk/medie/), Whatizit (http://www.ebi.ac.uk/webservices/whatizit/info.jsf), and BioIE (http://www.bioinf.manchester.ac.uk/dbbrowser/bioie/). These tools were applied in concert in order to redress some specific limitations of each individual approach (Fig 2A). Using this pipeline, ~1700 papers relating to “cardiac fibroblast” were extracted from PubMed.

Algorithm-based approaches to network reconstruction are, of course, susceptible to error (Fig 2A). In well-studied fields, a rich source of literature can be cross-compared to reconcile annotations and reduce error. However, as the literature on regulatory networks of CFb is sparse, mislabelling errors are more likely, and pose impediments to further research. To exploit the benefits of text mining without compromising accuracy, we extended the automated pipeline into a hybrid automated-manual approach (Fig 2B). The automated text mining analysis extracts the relevant body of literature from PubMed that is related to the CFb, and returns sentences containing highlighted biomedical terms. Then, manual curation is used to reconstruct the relationships among the biomedical terms and deduce the bio-relations. Following the automated text mining output, the manual curation process yielded ~1500 tagged bio-relations and ~650 bio-entities.

**Fig 3:**
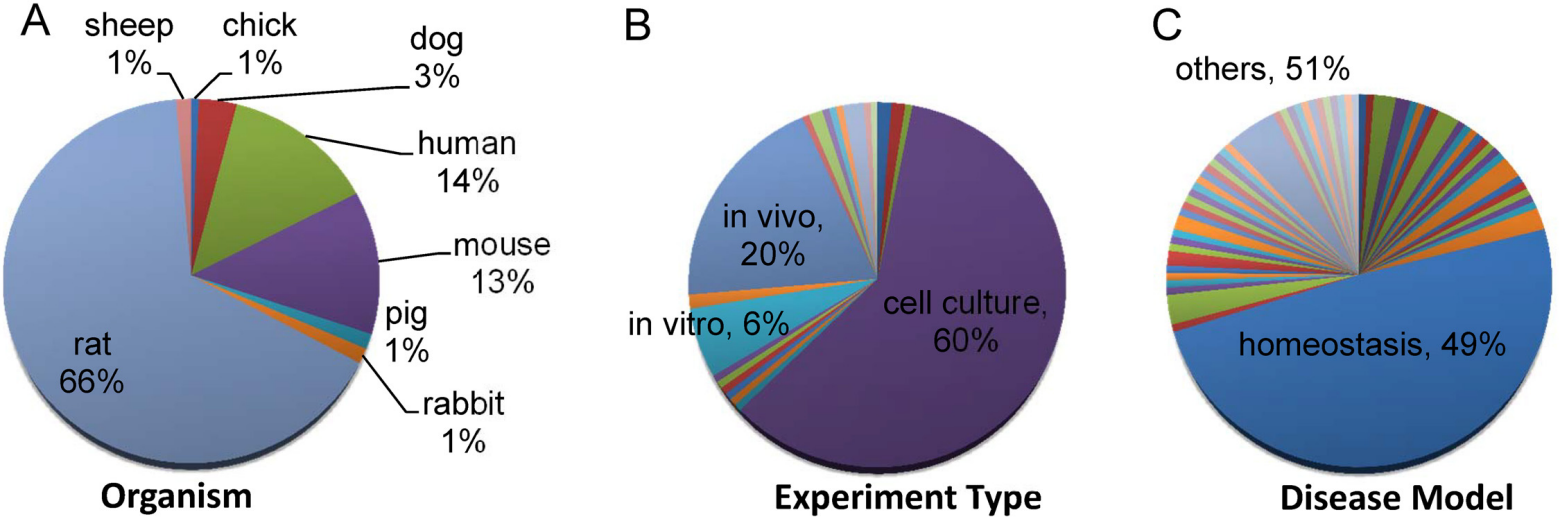
Manual statistical analysis of the literature collection process, as at the end of 2014. (A) Distributions of different organisms used in the experimental protocol. (B) Distribution of the techniques used in the experimental protocol. In this context, “*in vitro*” refers to the experiments performed on proteins and peptides in test tubes without cells; “cell culture” refers to the use of non-primary cell lines in the assays; and “*in vivo*” refers to studies that work on animal, tissue culture or primary cell culture. Other experimental protocols such as bioengineered tissue matrix are not labelled in the pie chart to improve readability. These labels can be found online in CARFMAP. Each article may contain more than one experimental protocol. (C) Distribution of the disease model being used in the experimental protocol. Homeostatic disease model refers to the experimental protocol involving healthy or normal tissues and cells. A myriad of non-homeostatic disease models were grouped as “others” to improve readability. More specific labels (such as cardiomyopathy, cardiac fibrosis) can be found online in CARFMAP. Each article may contain more than one disease model.

The collection of tagged sentences with labelled terms was then manually curated to transform the tagged bio-relations into one or more reaction-like events, such as where a bio-entity “activates” or “inhibits” another bio-entity. This provides the essential basis for constructing the cardiac fibroblast pathway map (CARFMAP). Manual curation also ensures the collection of the additional relevant tags related to experimental protocol, the organism being studied, and whether the system is a diseased or homeostatic model (Fig 3).

### Cardiac Fibroblast Interactive Pathway Map (CARFMAP)

**Fig 4:**
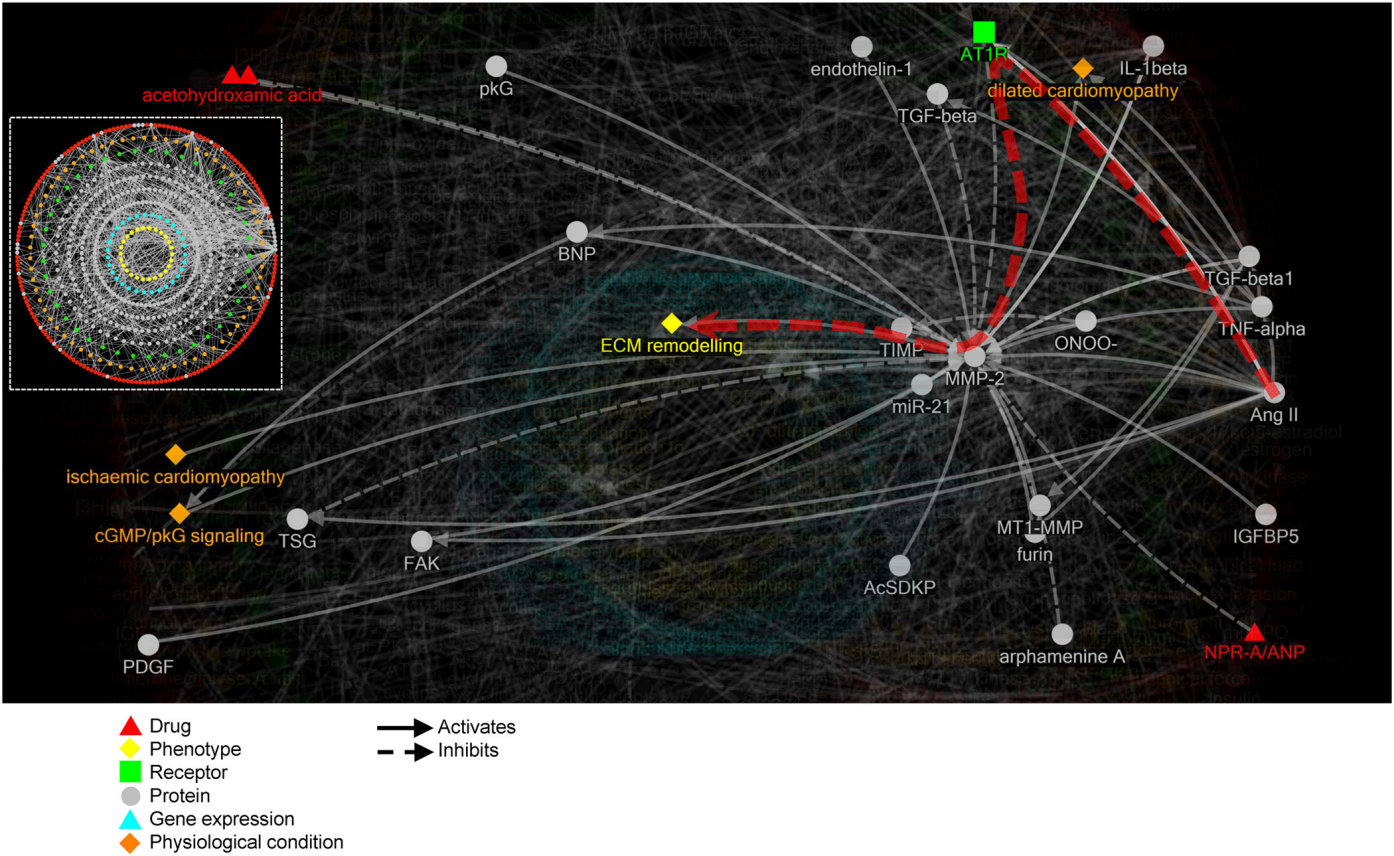
Illustration of CARFMAP graphical user interface, showing a sub-map comprising bio-entities connected to the “ECM remodelling” phenotype. Dashed white box (insertion) shows the map overview without node labels. Node colours encode the type of the bio-entities, and nodes are arranged in concentric rings, grouped by type. Protein (grey) nodes are divided into extra-cellular (outer grey ring) and intra-celluar (inner rings). Arrows indicate the bio-relations (“activates” or “inhibits”), and clicking each arrow will reveal the PubMed ID of the article citing this bio-relation. Arrow size is proportional to the number of articles citing the bio-relation (more citations generates a bolder arrow).

The annotated CFb data forms the building blocks of CARFMAP, with an interactive visualisation interface designed for biologist users (Fig 4). CARFMAP employs a polar layout that mimics the spatial distribution of the cellular components. Drugs (red), extracellular proteins (grey), and extracellular phenotypes (orange), are positioned as the 2 outermost rings. The next ring contains membrane receptors (green). Then several rings construct the intracellular proteins (grey), followed by gene expressions (cyan) and finally a central ring for the phenotypes (yellow). The bio-relations are represented using solid and dashed arrows, denoting “activates” and “inhibits” relationships, respectively.

Limited by the existing body of literature, CARFMAP does not yet cover every interaction in CFb, but already the map is too dense to be easily navigated. Thus, filters are available to highlight subsets of the pathway based on selected criteria, greatly improving human-readability. For example, the “extracellular matrix (ECM) remodelling” phenotype is used to highlight relevant nodes, and fade out irrelevant ones (Fig 4). Other types of filters are also implemented in CARFMAP, to facilitate visualisation of criteria of interest (S1 Fig). The most basic filter will display the information from one or several papers (S1A Fig). Next, filters can show only information from a particular experiment type (e.g. “*in vivo”*, S1B Fig), organism type (e.g. “mouse”, S1C Fig), or disease models (e.g. “homeostasis”, S1D Fig). Filters can also be combined to further zero in on a particular study in the literature, resulting in a combinatorial number of use cases for CARFMAP.

Any cardiac biologist who is interested in exploring the molecular and genetic mechanisms regulating this cell type can utilise the functionalities of CARFMAP. For example, if the interest is on how “Ang II” (angiotensin II) plays a role in “ECM (extra-cellular matrix) remodelling”, this pathway can be traced between these two bio-entities, revealing AT1R (angiotensin II receptor type 1) and MMP-2 (matrix metalloproteinase-2) as mediators of this function (Fig 4, thick red arrow). CARFMAP has a variety of use cases, and ultimately serves as a highly valuable interactive and specialised curated database, that will support ongoing cardiac research.

### Evaluating the integrity of CARFMAP and the literature base

CFb have a complex developmental origin and differentiation capacity [2, 3], and much debate still remains regarding the characteristic differences between CFb and other cell types such as mesenchymal stem cells, pericytes, *etc*. [18, 19]. Thus, the underlying literature from which CARFMAP is built upon needs to be broadly assessed in terms of quality and consistency. Also, while the articles collected for CARFMAP are CFb-related studies, the reported proteins and interactions may not be specific to only CFb.

**Fig 5:**
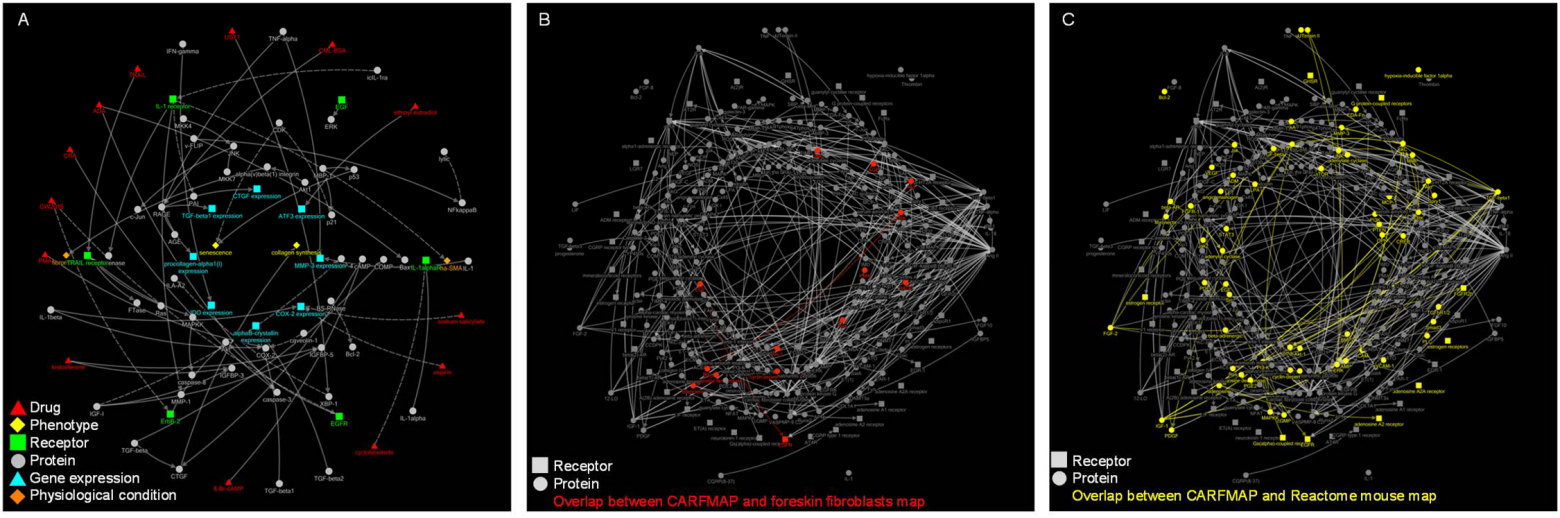
Integrity evaluation of CARFMAP. (A) Complete pathway map for foreskin fibroblasts. A total of 81 bio-entities and 63 bio-relations were collected from ~900 PubMed articles. (B) Overlap of proteins in CARFMAP and “foreskin fibroblasts”, with common nodes and edges highlighted in red. (C) Overlap of proteins in CARFMAP and a complete mouse pathway map from the REACTOME database, with common nodes and edges highlighted in yellow.

We therefore, we repeated the hybrid literature collection process for foreskin fibroblasts, another niche cell type, and a useful example for comparison to CARFMAP. The number of articles collected from PubMed (~900) was comparable to that for CARFMAP, but a major proportion (>80%) of these ~900 PubMed articles do not describe any bio-relation in our automated text mining analysis pipeline. These articles mainly adopt approaches such as epidemiology or clinical trials that do not describe regulatory relationships at the gene or protein levels. The collected bio-relations were converted into a second interactive map (Fig 5A) using the same approach as for CARFMAP. We then merged these maps and highlighted the common nodes and edges (Fig 5B). Overlapping nodes account for only <1% of the total nodes in CARFMAP.

Ideally, it would be interesting to derive a pathway for (all) fibroblasts in other mouse tissues, but there is currently no cell-type specific pathway database available, and it would be a mammoth undertaking with the currently available tools and literature resources. However, if one is looking for organism-wide signalling pathways, there are several online databases describing this information. We thus collected a massive signalling pathway for mouse from the REACTOME database [20] (S2 Fig). Surprisingly, we observed significantly little (<30%) overlap between CARFMAP and the REACTOME database (Fig 5C). Taken together, these results support the view that CFb-specific research is necessary to identify the important subset of genes and drug targets that are not being investigated in other cell types, and which are highly relevant to the field of heart research.

### Using CARFMAP to enhance gene expression profiles

**Fig 6:**
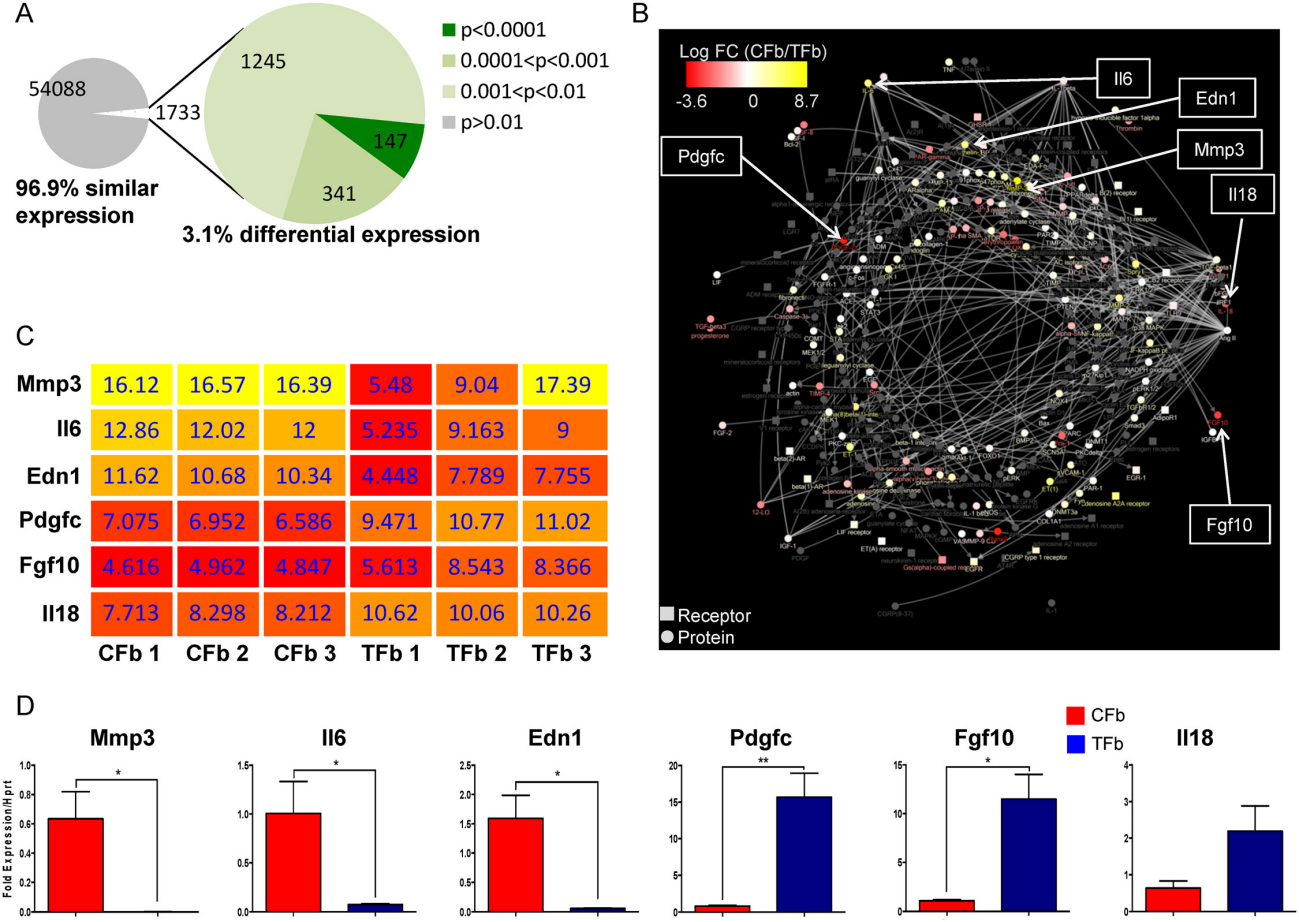
A case study of applying CARFMAP to facilitate cardiac gene discovery from expression profiles. (A) Pie chart summarising the *p*-value (unpaired *t*-test) of the normalised microarray dataset. 3.1% of the transcriptome has *p*-value < 0.01, which indicates differential expression between CFb and TFb. (B) CARFMAP overlaid with gene expressions of CFb and TFb. Node colours represents the log fold-change of the genes encoding the proteins and receptors. Note that only proteins and receptors are displayed. The log fold-change reveals CFb-specific genes that are strongly up-regulated or down-regulated with reference to TFb. (C) Heat map showing the differentially expressed genes from the microarray (with *p*<0.01) and CARFMAP. (D) qPCR validation of the candidate genes revealed by CARFMAP. Means and standard deviations (*n* = 3) are shown. (*): *p*<0.05; (**): *p*<0.005 (unpaired *t*-test).

Transcriptomic datasets are being generated at a rapid rate, including more than 30 data series about the CFb in the past 10 years. We previously profiled the expression levels for ~60,000 probes in CFb and TFb from healthy adult mice (GEO Accession: GSE50531) [11]. Interestingly, the microarray analysis revealed that CFb and TFb have 96.9% similar gene expression based on a *p*-value threshold of 0.01 (Fig 6A), leaving only a very small proportion of genes (3.1%) of high relevance to cardiac work.

Using our in-house visualisation platform [21], we assigned the log fold-change of the expression values between CFb and TFb as node colours (Fig 6B) in CARFMAP. The transcriptomic data can only be mapped to certain bio-entities (membrane receptors, proteins and gene expressions), so we filtered CARFMAP to omit other node types (drugs, phenotypes, *etc*.). This filtered visualization generates a list of 6 strongly up- and down-regulated gene candidates in CFb (Fig 6C). These candidate genes not only exhibit CFb-specific expression levels, their inclusion in CARFMAP is based on their frequent discussion in the highly specialised cardiovascular literature. Using qPCR, we validated these 6 differentially expressed genes. Although the gene *Il18* was not statistically significant (Fig 6D), the remaining 5 genes have been strongly associated with cardiovascular and other critical cellular functions (S1 Table), and further work may also reveal an important role for these genes in the context of the healthy adult mammalian heart.

## Discussion

The present study was based on the premise that the CFb plays a key role in maintaining heart homeostasis and disease, and that understanding the systems-level coordination between the CFb and other cardiac cell types is key to fully understanding the working heart. To the best of our knowledge, ours is the first effort to create a cardiac cell-specific pathway map, making CARFMAP a novel contribution to the cardiac research community. With the rapid advance of computational modelling of a whole cell [22], a blueprint of the current knowledge of the CFb is a timely and indispensible first step towards a systems-level understanding of this cell type. CARFMAP has been developed and specifically tailored to meet this need, and to bridge the technological strength of systems biology and the scientific strengths of cardiovascular research. CARFMAP not only serves as an essential step for systems-level investigation of the heart that integrates different cell types [19], it also encapsulates a new methodology and toolkit that facilitates the discovery of key genes within specific systems, that would otherwise be overlooked using the current conventional approaches.

Before proceeding with pathway perturbation experiments, it is essential to map out the state-of-art baseline knowledge, connecting the existing evidence into a unified conceptual framework. Most mechanistic models rely on accurate network structure [23], and manual curation is still the most reliable method for satisfying these constraints. Large pathway database systems (*e*.*g*. KEGG, REACTOME, Ingenuity IPA, MetaCore, *etc*.) have served as an invaluable resource [24, 25], facilitating many large-scale pathway modelling studies in recent years [26, 27]. However, information about cell-type specific pathways is not commonly available in these online repositories. We expect CARFMAP to be part of a growing effort to consolidate cell-type specific literature knowledge, and shed light on how hetero-cellular systems function. In the context of cardiac research, this will ultimately lead to a systems-level understanding of the functioning heart as a multicellular organ [28, 29], for concentrated efforts such as the Cardiac Physiome project [30], or the development of new (classes of) drugs.

In CARFMAP, we used broad category to describe bio-entities (“drugs”, “proteins”, “gene expression”) and bio-relations (“activates”, “inhibits”) to conservatively describe the underlying literature. There is always an inherent risk of false associations or description of the underlying biology from this text mining process, even with manual curation. As such, a typical cardiac biologist user of CARFMAP should look at a particular sub-pathway in CARFMAP using the filter function, then look up the relevant PubMed IDs to evaluate the integrity of the relationships behind it before proceeding any further.

In this study, we have limited our scope to the literature body of cardiac fibroblast to determine the baseline state of the heart as a heterocellular system [19]. While many studies are more concerned with the myofibroblasts potentially due to their clinical applications in cardiac fibrosis, our hypothesis is that the cardiac fibroblasts are actively working to maintain the heart homeostasis. While many of the collected articles describe myofibroblasts and the fibrosis disease model, only the bio-entities and bio-relations occurring in cardiac fibroblasts were included for CARFMAP. Some studies that purport to be on cardiac fibroblasts may indeed be on myofibroblasts due to the isolation and/or passaging schemes used [31]. Our cell isolation approach [9] where cells were freshly isolated, then plated for 5–7 days may also be subjected to this limitation. The heterogeneity of a CFb cell population is an important issue in our studies [9, 21] and this will greatly benefit from future development of better cell isolation techniques.

Pathways that are unique to CFb will serve as a fundamental characterisation tool for the cardiac research community. However, while CARFMAP describes signalling activities occurring in the context of CFb, as determined from the best available knowledge, it does not immediately follow that the information is CFb-specific. Thus, multiple essential integrity checks were applied to the collected data. Manual curation was used to identify and resolve differently labelled bio-entities (Fig 2). Statistical analysis of the literature generated an experimental context for the map by species, protocol, and disease state (Fig 3). Similar maps were generated for relevant other contexts, specifically foreskin fibroblasts, and the complete REACTOME mouse pathway map, and merged with CARFMAP (Fig 5). The 30% overlap between CARFMAP and REACTOME was surprising because we expected REACTOME (a massive pathway diagram) to contain most of the bio-relations in CARFMAP. Yet, Fig 5B shows that 70% of CARFMAP is not found in REACTOME, with some highly cardiac relevant bio-entities (such as Nkx2.5, GATA4, Tbx20) not found in any generic functional pathway described in REACTOME. The combination of these checks interestingly reveal that the bio-relations in CARFMAP do indeed appear to be quite specific to the cardiac research context, indicating that the field of CFb research is a niche area highly focused on a small number of bio-entities.

These findings were further reinforced when CARFMAP was integrated with the transcriptomes of CFb and TFb (Fig 6). Differential expression between mouse CFb and TFb can be used to outline the distinctive characteristics of the CFb. From this analysis, we identified a set of genes with marked differential expression levels, which supports the view that CFb is highly specialised for cardiac-specific functions. Note that visualisation of CARFMAP with overlaid gene expression colours (Fig 5C) does not automatically imply similar protein-level activities in the pathway. As proteomics technologies are becoming increasingly accessible, future work should continue to overlay CARFMAP with proteomics assays, similar to the transcriptomics of Fig 5C.

While there can be numerous use cases for CARFMAP, we selected and validated a popular and important scenario in which the map was overlaid with transcriptomic data to identify a set of distinguishing genes for the cardiac fibroblasts. Using a standard bioinformatics pipeline alone, researchers would have obtained between 147 and 1733 differentially expressed genes (Fig 6A, S2 Table), most of which have never been mentioned in the CFb literature. Our approach, including the literature curation as the basis for the data analysis, provided a set of candidate genes that may have otherwise been overlooked using a conventional experiment design approach, and our platform successfully identified 5 genes that have been well-discussed in the CFb research area, and could be validated in the laboratory. CARFMAP can additionally be used in conjunction with ChIP-Seq data for transcription factor analysis (S1 Appendix, S3 Fig). This demonstrates the ability of CARFMAP to facilitate parsing of the existing literature and to arrive at new biological insights with the aid of transcriptomic data integration.

It is important to note that the same discovery would not have been made using other high throughput analysis software. While there are currently many alternative databases containing pathway information (Ingenuity IPA, REACTOME, *etc*.), none of the existing databases contained cell type-specific interactions, particularly CFb-specific interactions. Among ~60,000 probes from the CFb-TFb microarray datasets, a standard bioinformatics-driven analysis could not single out the same five genes, regardless of the selection criteria (S2 Table). CARFMAP is tailored to cardiovascular research literature, and contains curated information through peer-reviewed publications, which underpins the successful use of the tool for cardiac research.

In any biological context, literature knowledge collection would greatly benefit from a comprehensive survey of the quality and quantity of the relevant literature bodies. In CARFMAP, we demonstrate that the quality of the literature body can be partially inferred based on the quantity of evidence, and the experimental protocol involved in each collected bio-relation. More importantly, CARFMAP provides the link to the associated literature to facilitate thorough investigation of the existing evidence surrounding the sub-map of interest. Users are not expected to accept it as baseline truth, but are encouraged to explore the articles via PubMed link, and consider the map in the context of the specific research under consideration. Among the CFb literature referenced in the process, most collected bio-relations were derived from laboratory-based rather than clinical-based research articles. From the article statistics (Fig 3), we observed an interesting phenomenon in the CFb literature: 66% articles describe experiments performed on rats and 60% articles describe experiments performed using cell culture. Finally, although manual curation has been performed to accurately reflect the literature information in the visual rendering, CARFMAP is only as accurate as the literature it represents. The pathway map represents a snapshot of the current literature related to CFb, as indexed by the NCBI PubMed database at the end of year 2014. This is a narrow scope, and further work must continue to characterise the CFb at the cell signalling level, which is more relevant to the distinctive phenotypes of the CFb and the understanding of its roles in the heart function. To account for this, we implemented CARFMAP in an updatable format, anticipating regular updates to incorporate newly published CFb articles. CARFMAP will be updated at least yearly to incorporate the ~100 and growing newly published articles each year.

The translational potential of the present study relies on the premise that CFb play a key role in maintaining heart homeostasis, and that the systems-level coordination between the CFb and other cardiac cell types is key to the understanding of the homeostatic heart. Aging-related heart attacks are often resistant to normal treatment with the drug statin [32], and this could be due to systems-level dysfunctions. To date only a handful of studies address the issue of aging or senescence in heart diseases [33–36], and the under-appreciated CFb may play a significant role towards a systems-level understanding of the heart [34, 37, 38].

With the development of CARFMAP, cardiac biologists can now explore the literature in a novel way, in the context of the (more realistic) complex network within which the component cells exist and function, rather than studying the genes or proteins individually, or treating all cell types as equal. This also opens the CFb field to a highly active research field of systems biology, where rapid advances have been made in the recent years. CARFMAP is developed based on cardiac biologists’ input, and therefore serves as a biologist-centric tool for experimental design.

## Supporting Information

S1 Appendix Transcription factor analyses based on ChIP-Seq and text mining. (PDF)

S1 Fig Filtering features and use cases for CARFMAP. Filters can be enabled in CARFMAP to show only bio-relations obtained (A) two PubMed articles; (B) experiments performed on Mouse; (C) experiments performed in vivo; and (D) experiments performed on homeostatic organisms.
(PDF)

S2 Fig REACTOME signal transduction pathway for *mus musculus*. SBML source file obtained by querying the REACTOME database for “*mus musculus*” and retrieving the most general pathway. The network was rendered by CellDesigner with the “organic layout” option.
(PDF)

S3 Fig TF network analysis to identify specific genes and TF for cardiac fibroblasts. (A) Transcription networks for two TFs: Gata6 and Hoxd8. Networks were constructed based on ChIP-Seq dataset, obtained from online databases (NCBI GEO, Stanford’s PRISM). Node colour indicates fold-change in expression between heart and tail fibroblasts. (B) Venn diagram showing the overlap between two TF networks (Gata6 and Hoxd8) or between 7 TF networks (Gata6, Gata4, Tbx20, Foxp2, Cdk8, Epas1, Hoxd8). (C) TF networks constructed based on literature mining (for genes with no available ChIP-Seq datasets). (D) Validation (using microarray expressions) of genes of interest from the experiment design pipeline. Means and standard deviation (n = 3) are shown.
(PDF)

S1 Table Functions of 5 genes revealed to be CFb-specific by CARFMAP [39–44]. (PDF)

S2 Table Standard differential analysis for Furtado et al.’s CFb-TFb microarray data [45]. The criterion that includes all 5 genes (AbsLogFC>2) and has the smallest number of genes (686) is highlighted in green.
(PDF)

## Abbreviations

Ang II: angiotensin II
AT1R: angiotensin II receptor type 1
BioIE: Extracting informative sentences from the biomedical literature
CFb: (the) cardiac fibroblasts
CM: cardiomyocytes
ECM: extracellular matrix
Edn1: endothelin 1
Fgf10: fibroblast growth factor 10
Gata4: GATA binding protein 4
Hprt: hypoxanthine-guanine phosphoribosyltransferase
Il18: interleukin 18
Il6: interleukin 6
IPA: Ingenuity Pathway Analysis
KEGG: Kyoto Encyclopedia of Genes and Genomes
MEDIE: Semantic retrieval engine for MEDLINE
MetaCore: Integrated pathway analysis for all omics data
MMP-2: matrix metalloproteinase-2
Mmp3: matrix metalloproteinase-3
Pdgfc: platelet-derived growth factor C
PubTator: a web-based tool for accelerating manual literature curation
REACTOME: an open-source, open access, manually curated and peer-reviewed pathway database
Tbx20: T-box transcription factor 20
TFb: (the) tail fibroblasts
Whatizit: text processing system from the European Bioinformatics Institute

## References

[1] Xin M, Olson EN, Bassel-Duby R. Mending broken hearts: cardiac development as a basis for adult heart regeneration and repair. Nature reviews Molecular cell biology. 2013;14(8):529–41. 10.1038/nrm3619 23839576 PMC3757945

[2] Souders CA, Bowers SL, Baudino TA. Cardiac fibroblast: the renaissance cell. Circulation research. 2009;105(12):1164–76. 10.1161/CIRCRESAHA.109.209809 19959782 PMC3345531

[3] Moore-Morris T, Tallquist MD, Evans SM. Sorting out where fibroblasts come from. Circ Res. 2014;115:602–4. 10.1161/circresaha.114.304854 . 25214570 PMC4176694

[4] Banerjee I, Fuseler JW, Price RL, Borg TK, Baudino TA. Determination of cell types and numbers during cardiac development in the neonatal and adult rat and mouse. Am J Physiol Heart Circ Physiol. 2007;293:H1883–91. 10.1152/ajpheart.00514.2007 . 17604329

[5] Chen W, Frangogiannis NG. Fibroblasts in post-infarction inflammation and cardiac repair. Biochimica et Biophysica Acta (BBA)—Molecular Cell Research. 2013;1833(4):945–53. 10.1016/j.bbamcr.2012.08.023 22982064 PMC3541439

[6] Snider P, Standley KN, Wang J, Azhar M, Doetschman T, Conway SJ. Origin of cardiac fibroblasts and the role of periostin. Circulation research. 2009;105(10):934–47. 10.1161/CIRCRESAHA.109.201400 19893021 PMC2786053

[7] Qian L, Srivastava D. Direct cardiac reprogramming: from developmental biology to cardiac regeneration. Circulation research. 2013;113(7):915–21. 10.1161/CIRCRESAHA.112.300625 24030021 PMC3869462

[8] Fu JD, Stone NR, Liu L, Spencer CI, Qian L, Hayashi Y, et al Direct reprogramming of human fibroblasts toward a cardiomyocyte-like state. Stem cell reports. 2013;1(3):235–47. Epub 12/10. 10.1016/j.stemcr.2013.07.005 eCollection 2013. . 24319660 PMC3849259

[9] Furtado MB, Costa MW, Pranoto EA, Salimova E, Pinto AR, Lam NT, et al Cardiogenic genes expressed in cardiac fibroblasts contribute to heart development and repair. Circ Res. 2014;114(9):1422–34. Epub 03/22. 10.1161/circresaha.114.302530 Epub 2014 Mar 20. . 24650916 PMC4083003

[10] Stennard FA, Costa MW, Elliott DA, Rankin S, Haast SJ, Lai D, et al Cardiac T-box factor Tbx20 directly interacts with Nkx2-5, GATA4, and GATA5 in regulation of gene expression in the developing heart. Developmental biology. 2003;262:206–24. Epub 10/11. . 14550786 10.1016/s0012-1606(03)00385-3

[11] Furtado MB, Nim HT, Gould JA, Costa MW, Rosenthal NA, Boyd SE. Microarray profiling to analyse adult cardiac fibroblast identity. Genomics Data. 2014;2(0):345–50. 10.1016/j.gdata.2014.10.006 26484127 PMC4536021

[12] Gentleman RC, Carey VJ, Bates DM, Bolstad B, Dettling M, Dudoit S, et al Bioconductor: open software development for computational biology and bioinformatics. Genome biology. 2004;5:R80 Epub 10/06. 10.1186/gb-2004-5-10-r80 . 15461798 PMC545600

[13] Smyth GK. limma: Linear Models for Microarray Data In: Gentleman R, Carey V, Huber W, Irizarry R, Dudoit S, editors. Bioinformatics and Computational Biology Solutions Using R and Bioconductor. Statistics for Biology and Health: Springer New York; 2005 p. 397–420.

[14] Rebholz-Schuhmann D, Oellrich A, Hoehndorf R. Text-mining solutions for biomedical research: enabling integrative biology. Nature reviewsGenetics. 2012;13(12):829–39. 10.1038/nrg3337 23150036

[15] Caron E, Ghosh S, Matsuoka Y, Ashton-Beaucage D, Therrien M, Lemieux S, et al A comprehensive map of the mTOR signaling network. Molecular systems biology. 2010;6:453 10.1038/msb.2010.108 21179025 PMC3018167

[16] Van Landeghem S, De Bodt S, Drebert ZJ, Inze D, Van de Peer Y. The potential of text mining in data integration and network biology for plant research: a case study on Arabidopsis. Plant Cell. 2013;25:794–807. Epub 03/28. Epub 2013 Mar 26. . 23532071 10.1105/tpc.112.108753 PMC3634689

[17] Poux S, Magrane M, Arighi CN, Bridge A, O'Donovan C, Laiho K. Expert curation in UniProtKB: a case study on dealing with conflicting and erroneous data. Database: the journal of biological databases and curation. 2014;2014:10. 10.1093/database/bau016 PMC3950660 24622611

[18] Krenning G, Zeisberg EM, Kalluri R. The origin of fibroblasts and mechanism of cardiac fibrosis. J Cell Physiol. 2010;225:631–7. Epub 07/17. 10.1002/jcp.22322 . 20635395 PMC3098503

[19] Nim HT, Boyd SE, Rosenthal NA. Systems Approaches in Integrative Cardiac Biology: Illustrations from Cardiac Heterocellular Signalling Studies. Progress in Biophysics and Molecular Biology. 2014: 10.1016/j.pbiomolbio.2014.11.006 25499442

[20] Joshi-Tope G, Gillespie M, Vastrik I, D'Eustachio P, Schmidt E, de Bono B, et al Reactome: a knowledgebase of biological pathways. Nucleic Acids Research. 2005;33(Database issue):D428–D32. 10.1093/nar/gki072 PMC540026. 15608231 PMC540026

[21] Nim HT, Furtado MB, Costa MW, Rosenthal NA, Kitano H, Boyd SE. VISIONET: intuitive visualisation of overlapping transcription factor networks, with applications in cardiogenic gene discovery. BMC Bioinformatics. 2015;16:141 Epub 05/02. 10.1186/s12859-015-0578-0 . 25929466 PMC4426166

[22] Karr JR, Sanghvi JC, Macklin DN, Gutschow MV, Jacobs JM, Bolival B, et al A whole-cell computational model predicts phenotype from genotype. Cell. 2012;150(2):389–401. 10.1016/j.cell.2012.05.044 22817898 PMC3413483

[23] Hyduke DR, Palsson BO. Towards genome-scale signalling network reconstructions. Nat Rev Genet. 2010;11:297–307. Epub 02/24. 10.1038/nrg2750 . 20177425

[24] Ng A, Bursteinas B, Gao Q, Mollison E, Zvelebil M. Resources for integrative systems biology: from data through databases to networks and dynamic system models. Brief Bioinform. 2006;7:318–30. Epub 10/17. . 17040977 10.1093/bib/bbl036

[25] Ghosh S, Matsuoka Y, Asai Y, Hsin KY, Kitano H. Software for systems biology: from tools to integrated platforms. Nature Reviews Genetics. 2011;12(12):821–32. 10.1038/nrg3096 22048662

[26] Gupta MK, Singh DB, Shukla R, Misra K. A comprehensive metabolic modeling of thyroid pathway in relation to thyroid pathophysiology and therapeutics. Omics: a journal of integrative biology. 2013;17:584–93. Epub 09/21. 10.1089/omi.2013.0007 Epub 2013 Sep 17. . 24044365

[27] Padwal MK, Sarma U, Saha B. Comprehensive logic based analyses of Toll-like receptor 4 signal transduction pathway. PLoS One. 2014;9:e92481 Epub 04/05. 10.1371/journal.pone.0092481 eCollection 2014. . 24699232 PMC3974726

[28] Hunter P, Nielsen P. A strategy for integrative computational physiology. Physiology (Bethesda, Md). 2005;20:316–25. Epub 09/22. 10.1152/physiol.00022.2005 . 16174871

[29] Kohl P, Noble D. Systems biology and the virtual physiological human. Mol Syst Biol. 5 England2009 p. 292 10.1038/msb.2009.51 19638973 PMC2724980

[30] Bassingthwaighte J, Hunter P, Noble D. The Cardiac Physiome: perspectives for the future. Experimental physiology. 2008;94:597–605. Epub 12/23. 10.1113/expphysiol.2008.044099 Epub 2008 Dec 19. . 19098089 PMC2854146

[31] Baum J, Duffy HS. Fibroblasts and myofibroblasts: what are we talking about? Journal of cardiovascular pharmacology. 2011;57(4):376–9. Epub 02/08. . 21297493 10.1097/FJC.0b013e3182116e39 PMC3077448

[32] de Jong HJ, Saldi SR, Klungel OH, Vandebriel RJ, Souverein PC, Meyboom RH, et al Statin-associated polymyalgia rheumatica. An analysis using WHO global individual case safety database: a case/non-case approach. PloS one. 2012;7(7):10. 10.1371/journal.pone.0041289 PMC3402515 22844450

[33] Anversa P, Leri A. Innate regeneration in the aging heart: healing from within. Mayo Clinic proceedings Mayo Clinic. 2013;88:871–83. Epub 08/06. 10.1016/j.mayocp.2013.04.001 . 23910414 PMC3936323

[34] Bujak M, Kweon HJ, Chatila K, Li N, Taffet G, Frangogiannis NG. Aging-related defects are associated with adverse cardiac remodeling in a mouse model of reperfused myocardial infarction. J Am Coll Cardiol. 2008;51:1384–92. Epub 04/05. 10.1016/j.jacc.2008.01.011 . 18387441 PMC3348616

[35] Camelliti P, Green CR, Kohl P. Structural and functional coupling of cardiac myocytes and fibroblasts. Advances in cardiology. 2006;42:132–49. Epub 05/02. 10.1159/000092566 . 16646588

[36] Ito N, Ohishi M, Yamamoto K, Tatara Y, Shiota A, Hayashi N, et al Renin-angiotensin inhibition reverses advanced cardiac remodeling in aging spontaneously hypertensive rats. Am J Hypertens. 2007;20:792–9. Epub 06/26. 10.1016/j.amjhyper.2007.02.004 . 17586415

[37] Gu C, Xing Y, Jiang L, Chen M, Xu M, Yin Y, et al Impaired Cardiac SIRT1 Activity by Carbonyl Stress Contributes to Aging-Related Ischemic Intolerance. PLoS One. 2013;8:e74050 Epub 09/17. 10.1371/journal.pone.0074050 . 24040162 PMC3769351

[38] Rando TA, Finkel T. Cardiac aging and rejuvenation—a sense of humors? The New England journal of medicine. 2013;369:575–6. Epub 08/09. 10.1056/NEJMcibr1306063 . 23924010

[39] Ye S, Eriksson P, Hamsten A, Kurkinen M, Humphries SE, Henney AM. Progression of coronary atherosclerosis is associated with a common genetic variant of the human stromelysin-1 promoter which results in reduced gene expression. The Journal of biological chemistry. 1996;271(22):13055–60. 8662692 10.1074/jbc.271.22.13055

[40] Wagner JA. Is IL-6 both a cytokine and a neurotrophic factor? The Journal of experimental medicine. 1996;183(6):2417–9. 8676061 10.1084/jem.183.6.2417 PMC2192601

[41] Ma LP, Premaratne G, Bollano E, Lindholm C, Fu M. Interleukin-6-deficient mice resist development of experimental autoimmune cardiomyopathy induced by immunization of β1-adrenergic receptor. International journal of cardiology. 2012;155(1):20–5. 10.1016/j.ijcard.2011.01.085 21334079

[42] Bruno CM, Neri S, Di P, Sciacca C. Pathophysiology of endothelin and medical emergencies. Panminerva medica. 2003;45(2):151–4. 12855940

[43] Choi SJ, Marazita ML, Hart PS, Sulima PP, Field LL, McHenry TG, et al The PDGF-C regulatory region SNP rs28999109 decreases promoter transcriptional activity and is associated with CL/Ps. European journal of human genetic: EJHG. 2009;17(6):774–84. 10.1038/ejhg.2008.245 19092777 PMC2788748

[44] Plichta JK, Radek KA. Sugar-coating wound repair: a review of FGF-10 and dermatan sulfate in wound healing and their potential application in burn wounds. Journal of burn care & research: official publication of the American Burn Association. 2012;33(3):299–310. 22561305 10.1097/BCR.0b013e318240540a PMC3348504

[45] Furtado MB, Costa MW, Pranoto EA, Salimova E, Pinto AR, Lam NT, et al Cardiogenic genes expressed in cardiac fibroblasts contribute to heart development and repair. Circulation research. 2014;114(9):1422–34. 10.1161/CIRCRESAHA.114.302530 24650916 PMC4083003

