# Supplementary material for: CARFMAP: A Curated Pathway Map of Cardiac Fibroblasts
Source: PLoS One. 2015 Dec 16;10(12):e0143274. doi: 10.1371/journal.pone.0143274 (PMC4684407; doi:10.1371/journal.pone.0143274)
Supplement: S1 Appendix — (PDF) [file pone.0143274.s001.pdf]

CARFMAP: a curated pathway map of cardiac fibroblasts

Hieu T. Nim[1,2], Milena B. Furtado[2], Mauro W. Costa[2], Hiroaki Kitano[2,3,4], Nadia A. Rosenthal[2,5,6] and Sarah E. Boyd[1,2]

1. Australian Regenerative Medicine Institute, Monash University, VIC 3800, Australia
2. Faculty of Information Technology, Monash University, VIC 3800, Australia
3. Laboratory for Disease Systems Modeling, RIKEN Center for Integrative Medical Sciences, Yokohama, Japan
4. Okinawa Institute of Science and Technology, Onna, Onna-son, Kunigami, Okinawa, Japan
5. National Heart and Lung Institute, Imperial College London, W12 0NN UK
6. The Jackson Laboratory, Bar Harbor, ME 04609, USA

S1 Appendix. Transcription factor analyses based on ChIP-Seq and text mining.

We denote "cardiac-specific" as genes having strongly up- or down-regulated expression in CFb, as compared to TFb. We used public ChIP-Seq datasets to identify the binding targets of cardiac-specific transcription factors. The overlap targets of two cardiac-specific TFs (Figure S3A) also include multiple cardiac-specific genes (strongly yellow or strongly red nodes). Replicating this process further, we identified 9 common targets of 7 cardiac-specific transcription factors (Figure S3B). Alternative to ChIP-Seq dataset, we also performed literature mining to collect the target genes of cardiac-specific TFs (Figure S3C). From this analysis, we collected 5 genes that are regulated by two or more TFs. It is also important to note that there are 5 cardiac-specific TFs that have no known binding targets from either ChIP-Seq database or the literature (orphans). The genes of interest are summarised in Figure S3D, which may serve as candidates for further experimental validation.
