# Supplementary figures and images for: CARFMAP: A Curated Pathway Map of Cardiac Fibroblasts
Source: PLoS One. 2015 Dec 16;10(12):e0143274. doi: 10.1371/journal.pone.0143274 (PMC4684407; doi:10.1371/journal.pone.0143274)

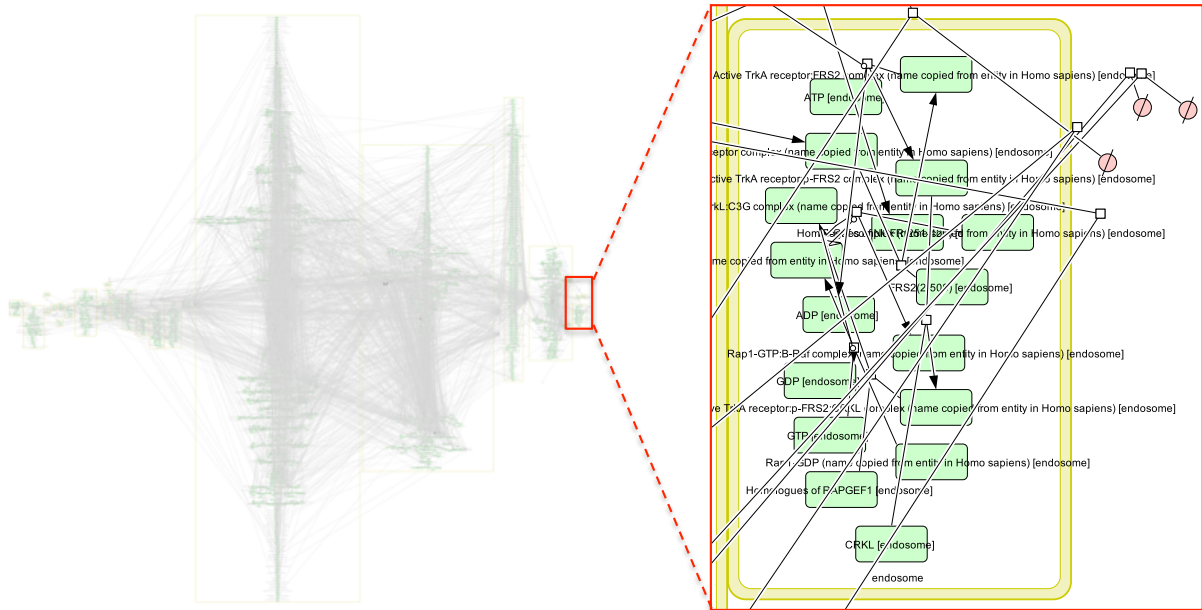

Supplement: S2 Fig — SBML source file obtained by querying the REACTOME database for “mus musculus” and retrieving the most general pathway. The network was rendered by CellDesigner with the “organic layout” option. (PDF) [file pone.0143274.s003.pdf]

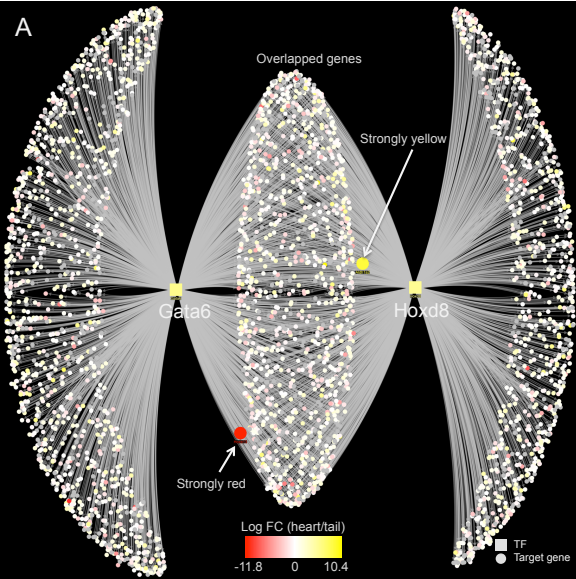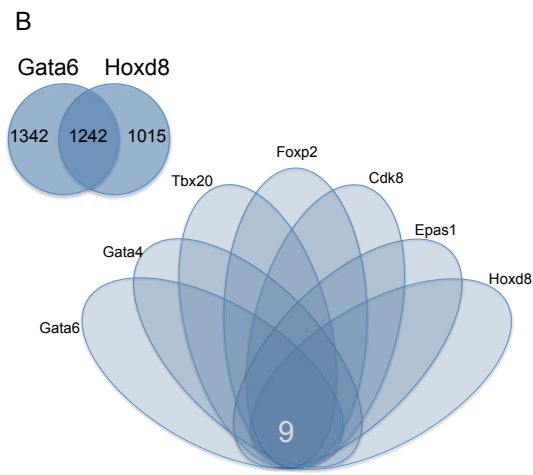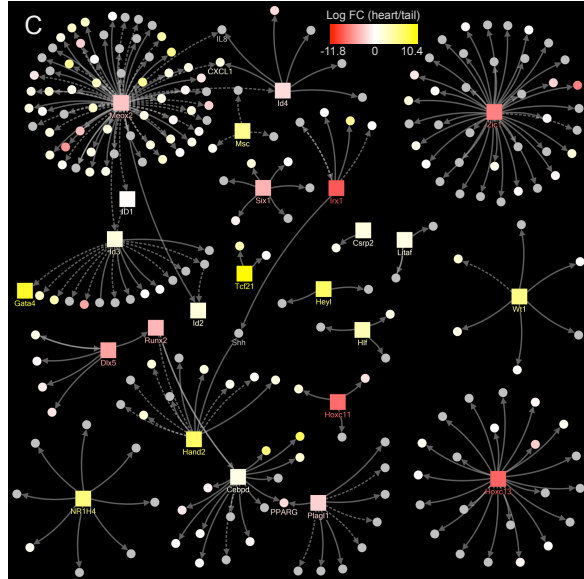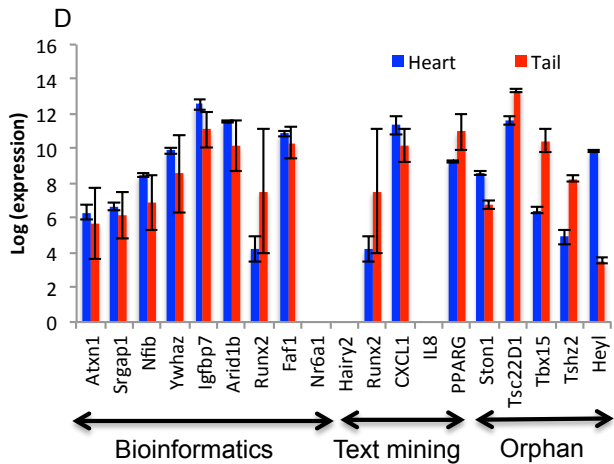

Supplement: S3 Fig — (A) Transcription networks for two TFs: Gata6 and Hoxd8. Networks were constructed based on ChIP-Seq dataset, obtained from online databases (NCBI GEO, Stanford’s PRISM). Node colour indicates fold-change in expression between heart and tail fibroblasts. (B) Venn diagram showing the overlap between two TF networks (Gata6 and Hoxd8) or between 7 TF networks (Gata6, Gata4, Tbx20, Foxp2, Cdk8, Epas1, Hoxd8). (C) TF networks constructed based on literature mining (for genes with no available ChIP-Seq datasets). (D) Validation (using microarray expressions) of genes of interest from the experiment design pipeline. Means and standard deviation (n = 3) are shown. (PDF) [file pone.0143274.s004.pdf]
