# Supplementary material for: CARFMAP: A Curated Pathway Map of Cardiac Fibroblasts
Source: PLoS One. 2015 Dec 16;10(12):e0143274. doi: 10.1371/journal.pone.0143274 (PMC4684407; doi:10.1371/journal.pone.0143274)
Supplement: S1 Table — (PDF) [file pone.0143274.s005.pdf]

**Genes**	**Functions**	**References**
Mmp3	extracellular matrix remodelling, collegen degradation	Ye 1996
Il6	inflammatory cytokine production, protein synthesis	Wagner 1996, Ma 2012
Edn1	vasocontriction	Bruno 2003
Pdgfc	palatogenesis, tissue morphogenesis	Choi 2009
Fgf10	embryonic development, cell growth, morphogenesis, tissue repair, tumor growth	Plichta 2012
