# Supplementary material for: CARFMAP: A Curated Pathway Map of Cardiac Fibroblasts
Source: PLoS One. 2015 Dec 16;10(12):e0143274. doi: 10.1371/journal.pone.0143274 (PMC4684407; doi:10.1371/journal.pone.0143274)
Supplement: S2 Table — The criterion that includes all 5 genes (AbsLogFC>2) and has the smallest number of genes (686) is highlighted in green. (PDF) [file pone.0143274.s006.pdf]

Criteria	Number of genes	Contain all 5 genes (Mmp3, Il6, Edn1, Pdgfc, and Fgf10)?
p<0.1	7626	Yes
p<0.01	1733	No
AbsLogFC>2	686	Yes
AbsLogFC>4	47	No
