# Supplementary material for: CARFMAP: A Curated Pathway Map of Cardiac Fibroblasts
Source: PLoS One. 2015 Dec 16;10(12):e0143274. doi: 10.1371/journal.pone.0143274 (PMC4684407; doi:10.1371/journal.pone.0143274)
Supplement: S1 Fig — Filters can be enabled in CARFMAP to show only bio-relations obtained (A) two PubMed articles; (B) experiments performed on Mouse; (C) experiments performed in vivo; and (D) experiments performed on homeostatic organisms. (PDF) [file pone.0143274.s002.pdf]

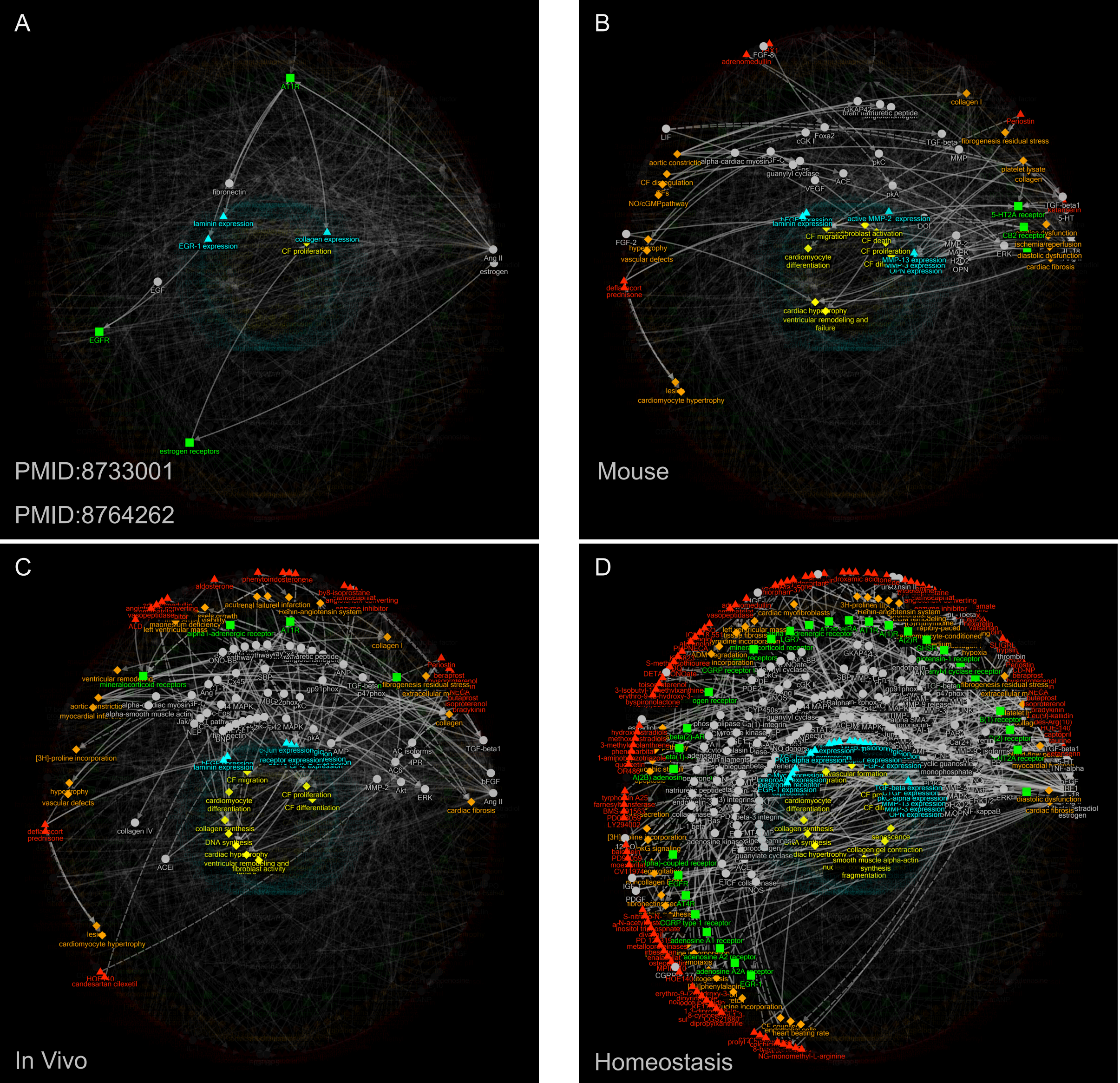
A
AT1R
fibronectin
laminin expression
EGR-1 expression
collagen expression
CF proliferation
Ang II
estrogen
EGF
EGFR
estrogen receptors
PMID:8733001
PMID:8764262
B
adrenomedullin
collagen I
Periostin
LIF
cGK I
Foxa2
TGF-beta
fibrogenesis residual stress
MMP
aortic constriction
guanylyl cyclase
ACE
pkC
pkA
VEGF
platelet lysate
collagen
NO/cGMPpathway
TGF-beta1
5-HT2A receptor
5-HT
FGF-2
hypertrophy
vascular defects
laminin expression
active MMP-2 expression
CF migration
CF death
CF proliferation
cardiomyocyte differentiation
MMP-13 expression
OPN expression
CB2 receptor
ischemia/reperfusion
diastolic dysfunction
cardiac fibrosis
ERK
MMP-2
MAPK
H2O2
OPN
deflazacort
prednisone
cardiac hypertrophy
ventricular remodeling and failure
cardiomyocyte hypertrophy
Mouse
C
aldosterone
phenytoindosterone
AT1R
alpha1-adrenergic receptor
left ventricular mass
collagen I
Periostin
ventricular remodeling
mineralocorticoid receptors
TGF-beta
fibrogenesis residual stress
p47phox
gp91phox
extracellular matrix
isoproterenol
bradykinin
collagen
myocardial infarction
alpha-smooth muscle actin
[3H]-proline incorporation
TGF-beta1
laminin expression
c-Jun expression
AC isoforms
IPR
AC6
MMP-2
Akt
ERK
bFGF
Ang II
cardiac fibrosis
hypertrophy
vascular defects
CF migration
cardiomyocyte differentiation
CF proliferation
CF differentiation
deflazacort
prednisone
collagen IV
collagen synthesis
DNA synthesis
cardiac hypertrophy
ventricular remodeling and
fibroblast activity
ACEi
cardiomyocyte hypertrophy
candesartan cilexetil
In Vivo
D
adrenomedullin
cardiac myofibroblasts
Renin-angiotensin system
AT1R
collagen synthesis
senescence
smooth muscle alpha-actin
collagen gel contraction
adenosine A1 receptor
adenosine A2 receptor
adenosine A2A receptor
EGFR
AT4R
CGRP type 1 receptor
diastolic dysfunction
cardiac fibrosis
estradiol
estrogen
heart beating rate
NG-monomethyl-L-arginine
Homeostasis
